# Genetic Diversity of Upland Rice Germplasm in Malaysia Based on Quantitative Traits

**DOI:** 10.1100/2012/416291

**Published:** 2012-04-30

**Authors:** M. Sohrabi, M. Y. Rafii, M. M. Hanafi, A. Siti Nor Akmar, M. A. Latif

**Affiliations:** ^1^Institute of Tropical Agriculture, Universiti Putra Malaysia, 43400 Serdang, Selangor, Malaysia; ^2^Department of Crop Science, Faculty of Agriculture, Universiti Putra Malaysia, 43400 Serdang, Selangor, Malaysia; ^3^Bangladesh Rice Research Institute (BRRI), Gazipur 1701, Bangladesh

## Abstract

Genetic diversity is prerequisite for any crop improvement program as it helps in the development of superior recombinants. Fifty Malaysian upland rice accessions were evaluated for 12 growth traits, yield and yield components. All of the traits were significant and highly significant among the accessions. The higher magnitudes of genotypic and phenotypic coefficients of variation were recorded for flag leaf length-to-width ratio, spikelet fertility, and days to flowering. High heritability along with high genetic advance was registered for yield of plant, days to flowering, and flag leaf length-to-width ratio suggesting preponderance of additive gene action in the gene expression of these characters. Plant height showed highly significant positive correlation with most of the traits. According to UPGMA cluster analysis all accessions were clustered into six groups. Twelve morphological traits provided around 77% of total variation among the accessions.

## 1. Introduction

Around 3 billion people of the world use rice as a critical or basic food that provides 50 to 80% of their daily calories. Rice is cultivated on more than 150 million hectares, and annual world production is around 600 million tons [1–3]. Upland rice comprises eleven percent of global rice production and is cultivated on around 14 million hectares. Upland rice has a small role in total production but is major food in some tropical countries [4]. Bangladesh, Indonesia, and Philippines are the areas that plant the most upland rice, but the yield is so low (about 1 t/ha on average) and highly variable [5, 6].

In Malaysia, two types of rice are cultivated: wetland rice in Peninsular Malaysia (503,184 ha) and upland rice in Sabah and Sarawak (165,888 ha). The average yield of wetland rice is around 3.3 t/ha; in good conditions, however, it can increase to around 10 t/ha. In contrast, the average yield of upland rice ranges from 0.46 to 1.1 t/ha. In 2005, the total national rice production was roughly 2.24 million metric tons. In Malaysia, upland rice is usually cultivated for home consumption by rural people living in Sabah and Sarawak [7].

Genetic diversity is the basis of plant breeding, so understanding and assessing it is important for crop management, crop improvement by selection, use of crop germplasm, detection of genome structure, and transfer of desirable traits to other plants [8, 9]. Rice is one of the best plants for the study of genome structure and genetic diversity because it is diploid and has a small genome size of 430 Mb [10], a significant level of genetic polymorphism [11, 12], and a large amount of well-conserved genetically diverse material.

The breeders are interested to evaluate genetic diversity based on morphological traits because they are inexpensive, rapid, and simple to score. The study of these traits needs neither sophisticated methods nor complicated equipments, and also these traits can be inherited without either specific biochemical or molecular techniques. Until now scientific classification of plant was based on morphological traits [13, 14]. The rice plant (*Oryza sativa*) shows great morphological variation, especially in vegetative traits such as plant height and leaf length. Therefore, the present study was undertaken to assess the genetic diversity of upland rice genotypes in Malaysia.

## 2. Materials and Methods

### 2.1. Plant Material and Experimental Design

Fifty accessions of upland rice were selected from MARDI (24 from Peninsular Malaysia and 26 from Sabah). The accessions were cultivated in experimental field of Universiti Putra Malaysia. Sprouted seeds were sown in the pots (Table 1). Randomized complete block design (RCBD) with three replications was used with 50 pots for each replication.

### 2.2. Data Collection

Twelve quantitative traits were recorded for all accessions at each replication: plant height (cm), days to flowering (day), days to maturity (day), flag-leaf-length-to-width ratio (cm), number of tillers per hill (no.), number of grains per panicle (no.), one thousand grains weight (g), yield of plant per pot (g), number of panicles per hill (no.), panicle length (cm), spikelet per panicle (no.), and spikelet fertility (%) (Table 2).

### 2.3. Statistical Analysis

The analysis of variance (ANOVA) revealed the main interaction effects. Least significant difference (LSD) was calculated using Statistical analysis system software (SAS version 9.1) (Table 3). Genetic parameters were estimated to identify genetic variation among accessions and to determine genetic and environmental effects on various characters. These genetic parameters were calculated by the formula given by Burton [15], Burton and De Vane [16], and Johnson et al. [17]. These parameters include the following.

- Genotypic variance:
  (1) $$\sigma_{g}^{2}=\frac{\text{MSG}-\text{MSE}}{r},$$
  where MSG is the mean square of accessions, MSE is mean square of error, and *r* is number of replications.
- Phenotypic variance:
  (2) $$\sigma_{p}^{2}=\sigma_{g}^{2}+\sigma_{e}^{2},$$
  where *σ*_*g*_^2^ is the genotypic variance and *σ*_*e*_^2^ is the mean squares of error.
- Phenotypic coefficient of variance (PCV):
  (3) $$\text{PCV}\left(\text{\%}\right)=\frac{\sqrt{\sigma_{p}^{2}}}{\overset{®}{X}}\times 100,$$
  where *σ*_*p*_^2^ is the phenotypic variance and $\overset{®}{X}$ is the mean of trait.
- Genotypic coefficient of variance (GCV):
  (4) $$\text{GCV}\left(\text{\%}\right)=\frac{\sqrt{\sigma_{g}^{2}}}{\overset{®}{X}}\times 100,$$
  where *σ*_*g*_^2^ is the genotypic variance and $\overset{®}{X}$ is the mean of character.
- Heritability (broad sense):
  (5) $$h_{B}^{2}=\frac{\sigma_{g}^{2}}{\sigma_{p}^{2}},$$
  where *σ*_*g*_^2^ is the genotypic variance and *σ*_*p*_^2^ is the phenotypic variance.
- Expected genetic advance (GA):
  (6) $$\text{GA}\left(\text{\%}\right)=K\times \sigma_{p}^{2}\times h_{B}^{2}\times 100.$$
  GA is a percent of the mean assuming selection of the superior 5% of accession:
  (7) $$\text{GA}\left(\text{\%}\right)=K\times \frac{\sqrt{\sigma_{p}^{2}}}{\overset{®}{X}}\times h_{B}^{2}\times 100,$$
  where *K* is a constant,$\;\sqrt{\sigma_{p}^{2}}/\overset{®}{X}$ is the phenotypic standard deviation,  *h*_*B*_^2^ is the heritability, and$\;\overset{®}{X}$ is the mean of traits.

The correlation coefficient was analyzed to evaluate the relationships among the different variables in the experiment using SAS software (version 9.1). Data were also analyzed based on Jaccard*ʼ*s similarity coefficient by NTSYS-pc software (version 2.1). UPGMA algorithm and SAHN clustering were applied for calculating genetic relationships. The PCA of fifty accessions was calculated by EIGEN and PROJ modules of NTSYS-pc and Minitab software (version 15).

## 3. Result

### 3.1. Variation and Genetic Parameters among Accessions

Eight traits including plant height, days to flowering, flag leaf length-to-width ratio, 1000-GW, yield per pot, panicle length, spikelet per panicle, and spikelet fertility showed highly significant (*P* ≤ 0.01) variation and the rest of them such as days to maturity, number of tillers per hill, number of grains per panicle, and number of panicles per hill were significant (*P* ≤ 0.05) among all accessions (Table 4).

In this study, most of the growth traits showed higher PCV compared to yield and yield component traits. However, lower PCV belonged to plant height (15.85%) while flag leaf length-to-width ratio (69.63%) was recorded as higher value. Spikelet fertility (47.31%), days to flowering (40.94%), and days to maturity (40.77%) were recorded as higher values of PCV and number of grains per panicles (21.27%), number of panicle (24.54%), and panicle length (24.63%) showed lower values. The higher GCV was recorded at flag leaf length-to-width ratio (66.66%) and the lower was found at plant height (14.92%). GCV value was low in yield and yield components compared to growth characters. Board sense heritability ranged from 60.26 to 99.84%. The highest and the lowest amount of heritability was recorded at yield of plant and number of panicles, respectively. The estimates of heritability were high for 1000 GW (99.76%), spikelet fertility (94.08%), panicle length (91.69%), flag leaf length-to-width ratio (91.63%), plant height (88.57%), days to flowering (85.54%), spikelet per panicle (81.35%), and days to maturity (80.28%) whereas other characters showed relatively low heritability. GA ranged from 28.93% for plant height to 131.45% for flag leaf length-to-width ratio. The average of GA value in growth traits was higher than the average of GA value in yield and yield components (Table 5).

### 3.2. Association between Traits

Pearson's correlation coefficient was computed between 12 quantitative traits among 50 accessions of upland rice (Table 6). Positive correlation was found between most of traits. Plant height was highly significant and positively correlated with most of traits such as days to flowering, days to maturity, flag leaf width-to-length ratio, number of grains per panicle, yield of plant, panicle length, and spikelet fertility. Yield of plant had highly significant (*P* < 0.01) and positively correlated with plant height (*r* = 0.38), days to maturity (*r* = 0.36), and number of panicles (*r* = 0.48) at 1% probability level and also significant (*P* ≤ 0.05) and positively correlated with days to flowering (*r* = 0.31) and 1000-grain weight (*r* = 0.34).

### 3.3. Cluster Analysis

**Figure 1:**
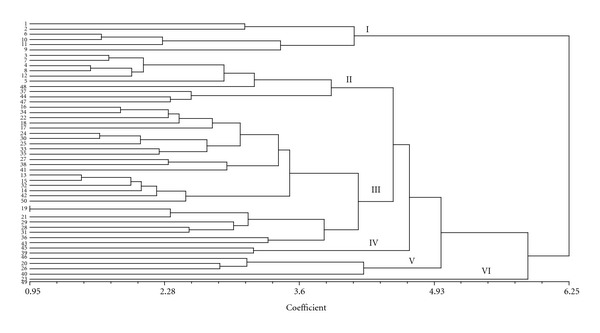
The dendrogram of 50 accessions of upland rice based on 12 quantitative traits.

Fifty accessions of upland rice were clustered into six groups by 12 quantitative traits. As evident from Figure 1 and Table 7 cluster III was the biggest (27 accessions) and cluster VI was the smallest (only one member) group. Cluster I, II, IV, and V consisted of 6, 10, 2, and 4 members, respectively. The first group had the highest average in comparison with the other five groups considering five traits (Table 8) such as plant height (147.9 cm), days to flowering (112.8 days), days to maturity (144 days), flag leaf length-to-width ratio (31.1 cm), and panicle length (30.07 cm). Group VI included the highest average for four traits such as number of tillers (4.7), 1000 GW (33 g), yield of plant (55.1 g), and spikelet fertility (95.8%). On the other hand, accessions having this group (VI) showed the lowest average values in the characters such as plant height, days to maturity, flag leaf length-to width ratio, number of panicles, panicle length, and spikelet per panicle.

### 3.4. Principal Component Analysis (PCA)

**Figure 2:**
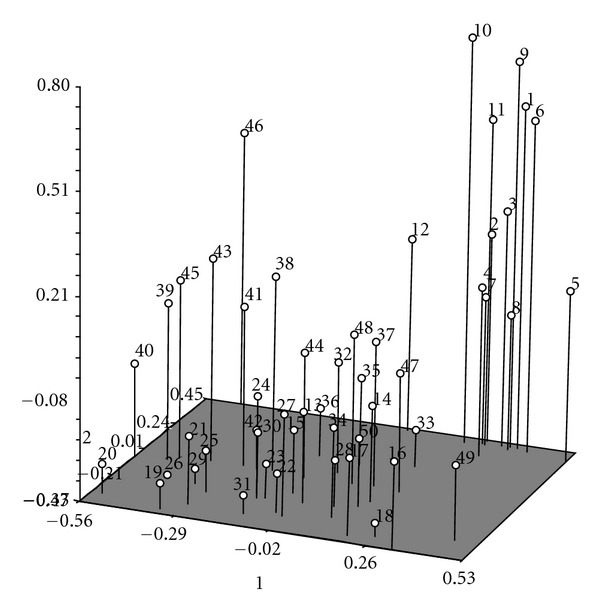
Three-dimensional graph of 50 upland rice accessions based on 12 quantitative traits.

PCA approximately confirmed the cluster analysis for distant accession, 07508, and it was clustered alone in cluster VI (Figure 2). But on the other hand, some accessions were close together in PCA such as accessions 06040, 06041, 06070, 06067, 06043, 06050, and 06059, whereas they were clustered into 2 groups (group I and II) in cluster analysis. According to PCA, the first four principal components accounted for around 76.7% of total variation of all morphological traits. The analysis of eigenvectors indicated the information of morphological traits for percentage of variation to the first four principal components, which were 36.4, 17.9, 12.8, and 9.6%, respectively (Table 9).

## 4. Discussion

All traits showed highly significant (*P* < 0.01) and significant (*P* < 0.05) variations among 50 accessions, which originated in Peninsular Malaysia and Sabah. Pandey et al. [18] recorded highly significant difference among 40 genotypes of rice with 12 quantitative traits. Wang et al. [19] observed 95% differentiation among 5 populations of rice by 20 morphological traits. Caldo et al. [20] measured highly significant difference (*P* < 0.01) in 41 morphological characteristics between 81 ancestors of rice and also CV ranged from 2.0% for grain length, grain width, and 1000 grains weight to 22.1% for culm number. Chandra et al. [21] and Abarshahr et al. [22] measured highly significant variation at 0.01 revealed by 14 and 19 quantitative traits among 57 accessions of upland rice and 30 genotypes of rice, respectively.

Correlation between traits is so important because it helps the breeder to select important characters from the studied traits. Most of the traits such as yield and yield component traits are influenced by interaction of genotype and environment, and, therefore, selection based on correlation coefficient makes it easy for plant breeders [23]. As mentioned, in this assay yield of plant had positive correlation with 12 morphological traits. Lasalita-Zapico et al. [24] evaluated correlation coefficient of 10 quantitative traits for 32 upland rice varieties. In this distinguish significant positive correlation among the majority of the morphological traits was recorded except flag leaf angle that had negative correlation with most of characters such as panicle length, leaf length, leaf width, ligule length, leaf area, and culm length. Zafar et al. [25] recorded positive correlation coefficient of panicle length (yield component) with tillers of plant and 100 grains weight and also significantly positive correlation with grain length (0.278).

The computing of heritability and genetic advance useful for selection on phenotypic expression [17]. Therefore, high amount of heritability and genetic advance can be the base of selection according to morphological traits. In present study, flag leaf length-to-width ratio, spikelet fertility, yield of plant, and days to flowering indicated both high heritability and genetic advance. Thus, selection based on these traits would bring about improvement in the genotypes. In previous studies, Sedeek et al. [26] reported both high heritability and high genetic advance for days to heading, flag leaf area, number of filled grains per panicle, and grain yield per plant. The heritability ranged between 86% and 99.4%, and for genetic advance was ranged from 17.81% for number of panicles per plant to 46.16% for grain yield per plant among 24 of rice varieties. Laxuman et al. [27] recorded high heritability (more than 60%) and high genetic advance (more than 20%) for chlorophyll meter reading, number of productive tillers per plant, panicle weight and number of grains per panicle, and 1000 grain weight. Pandey et al. [18] recorded high broad sense heritability among 40 rice varieties for plant height (99.8%), biological yield (99.6%), harvest index (99%), test weight (98.8 g), number of panicles per hills (98.5%), number of spikelets per panicle (98.3%), and grain yield (98.11 g).

In our study, fifty accessions of the upland rice were clustered into six groups based on 12 quantitative traits. Ahmadikhah et al. [23] clustered 58 rice varieties into four groups based on 18 morphological traits, and genetic distance was around 0.75. Group A composed of only one member and groups B, C, and D contained 14, 20, and 23 members, respectively. Veasey et al. [28] computed clustering for 23 populations of rice by 20 morphological characteristics. So the varieties were clustered into 10 groups of the last group was the biggest group with seven members and groups 1, 2, 7, and 8 were the smallest groups including only one variety. So, genotypes having distant clusters could be hybridized to get the higher heterotic responses. The similar studies were reported by several authors [29–31].

Principal component analysis indicated diversity among 50 accessions of upland rice by a few eigenvectors. In the present study, the first four principal components indicated 76.4% of total variation for which PC1 showed 36.4% of the variation PC2, PC3, and PC4 explained 17.9%, 12.8%, and 9.6% of total variation, respectively. Lasalita-Zapico et al. [24] computed approximately 82.7% of total variation among 32 upland rice varieties, 66.9% variation for PC1 and 15.87% for PC2. Caldo et al. [20] recorded the first 10 principal components accounting for 67% of total variation. This suggested a strong correlation among characters being examined. Rajiv et al. [32] reported the first two principal components accounting for 82.1% of total variation in control and 68.6% in the stress-induced genotypes.

## 5. Conclusion

Fifty accessions of upland rice were clustered into six main groups. To achieve a wide spectrum of variation among the segregates, genotypes having distant cluster, group I (accessions 6040, 6041, 6048, 6068, 6070, and 6067) could be hybridized with group V (accessions 7541, 7596, 3828, and 7545) and group VI (accession 7508). Principal component analysis indicated 76.4% of the total variation. PCA and cluster analysis complemented each other with some slight inconsistencies in terms of cluster composition. Heritability is one of the most important factors in statistical analysis. Separation and selection of varieties based on high heritability of traits make it easy for breeders. Most researchers agree that high heritability alone is not enough; both high heritability and high genetic advance are needed. In this experiment, flag leaf length-to-width ratio, spikelet fertility, yield of plant, and days to flowering had high heritability and high genetic advance. Most traits such as plant height, yield of plant, panicle length, number of panicles, and days to flowering had positive correlations among each other, which suggested that utilization of these traits could improve the genotype by selection of desirable varieties.

## Figures and Tables

**Table 1:** Information on locations, seasons of seed collection and local name of the upland rice accessions.

SL	Accessions	Location	Season	Local name
1	6040	Peninsular Malaysia	Main season (MS)	Bedor
2	6041	Peninsular Malaysia	Off season (OS)	Berjer
3	6043	Peninsular Malaysia	Main season (MS)	Buih
4	6044	Peninsular Malaysia	Main season (MS)	Gemalah
5	6045	Peninsular Malaysia	Off season (OS)	Kura
6	6048	Peninsular Malaysia	Main season (MS)	Piya
7	6050	Peninsular Malaysia	Off season (OS)	Ulat
8	6059	Peninsular Malaysia	Off season (OS)	Rengan bembang
9	6067	Peninsular Malaysia	Main season (MS)	Lumut/Kuku balam
10	6068	Peninsular Malaysia	Main season (MS)	Padi Kuku balam
11	6070	Peninsular Malaysia	Main season (MS)	Selayang
12	6071	Peninsular Malaysia	Main season (MS)	Lalang
13	7531	Sabah	Main season (MS)	Kungkuling A
14	7534	Sabah	Main season (MS)	Bukit
15	7535	Sabah	Main season (MS)	Pagalan
16	7537	Sabah	Main season (MS)	Sibuku
17	7538	Sabah	Main season (MS)	Lapaung
18	7539	Sabah	Main season (MS)	Sanding
19	7540	Sabah	Main season (MS)	Putus tunang
20	7541	Sabah	Main season (MS)	Ruabon
21	7543	Sabah	Main season (MS)	Semiali
22	7544	Sabah	Main season (MS)	Tadaong
23	7545	Sabah	Main season (MS)	Tayakon kecil
24	7546	Sabah	Main season (MS)	Teun
25	7597	Sabah	Main season (MS)	Batangan
26	7596	Sabah	Main season (MS)	Kaca
27	7595	Sabah	Main season (MS)	Turayo
28	7594	Sabah	Main season (MS)	Tarakan
29	7590	Sabah	Main season (MS)	Dinabor
30	7589	Sabah	Main season (MS)	Rangayat
31	7588	Sabah	Main season (MS)	Turakin
32	7585	Sabah	Main season (MS)	Peturu
33	7576	Sabah	Main season (MS)	Pagalan
34	7571	Sabah	Main season (MS)	Turayan
35	7574	Sabah	Main season (MS)	Dedawar
36	7575	Sabah	Main season (MS)	Lelangsat
37	3824	Peninsular Malaysia	Main season (MS)	Huma kuning lenggong
38	3825	Peninsular Malaysia	Main season (MS)	Huma wangi lenggong
39	3826	Peninsular Malaysia	Off season (OS)	Jarom mas
40	3828	Peninsular Malaysia	Main season (MS)	Kunyit
41	3830	Peninsular Malaysia	Main season (MS)	Langsat
42	3831	Peninsular Malaysia	Off season (OS)	Lenggong
43	3832	Peninsular Malaysia	Main season (MS)	Puteh perak
44	3834	Peninsular Malaysia	Off season (OS)	Rambut
45	3833	Peninsular Malaysia	Off season (OS)	Putih
46	3837	Peninsular Malaysia	Main season (MS)	Tangkai langsat
47	3838	Peninsular Malaysia	Main season (MS)	Wangi puteh
48	3835	Peninsular Malaysia	Off season (OS)	Rengan wangi
49	7508	Sabah	Main season (MS)	Beliong
50	7509	Sabah	Main season (MS)	Bedumpok

**Table 2:** List of quantitative traits of upland rice.

Traits	Method of evaluation
Plant height (PH, cm)	The average of height from the base to the tip of last leaf (flag leaf)
Days to flowering (DF, days)	The number of days from seeding to flowering day
Days to maturity (DM, days)	The number of days from seeding to maturing day
Flag leaf length to width ratio (FLR, cm)	Dividing the flag leaf length to width
Number of tillers per hill (NT, no.)	Counting of the tillers per hill
Number of grains per panicle (NG, no.)	Counting the number of grains on per panicle
One thousand grain weight (1000 GW, g)	200 grains were weighted then 1000 weight grains were calculated from these weights
Yield of plant per pot (YP, g)	Weighting total grains per pot
Number of panicles per hill (NP, no.)	Counting the panicles per hill
Panicle length (PL, cm)	From base of the lowest spikelet to the top of latest spikelet on panicle
Spikelet per panicle (SP, no.)	Counting the spikelet per panicle
Spikelet fertility (SF, %)	Dividing ripped spikelet to all spikelet

**Table 3:** ANOVA showing source of variation, degrees of freedom, means square, and error mean square.

Source of variation	df	MS	EMS
Blocks (*r*)	*r* − 1	MSB	*σ*_*e*_^2^ + *gσ*_*r*_^2^
Accessions (*g*)	*g* − 1	MSG	*σ*_*e*_^2^ + *rσ*_*g*_^2^
Groups (*t*)	[*t* − 1]	MST	*σ*_*e*_^2^ + *rσ*_*g*/*t*_^2^ + *r* *gσ*_*t*_^2^
Accessions/groups	[*t*(*g* − 1)]	MSG/T	*σ*_*e*_^2^ + *rσ*_*g*/*t*_^2^
Error	(*r* − 1)(*g* − 1)	MSE	*σ*_*e*_^2^

*r*: blocks, *g*: accessions, *t*: groups, *e*: error, df: degree of freedom, MS: mean squares, EMS: expected mean squares.

**Table:** (a) Mean squares of analysis of variance for 5 traits among 50 accessions of upland rice.

Source of variation	df	PH	DF	DM	FLR	NT
Blocks	2	0.01^ns^	6.61^ns^	41.33^ns^	0.01^ns^	0.0027^ns^
Accessions	49	1295.77**	3236.17**	5410.90*	617.37**	2.12*
Groups	[1]	4977.62**	15272.12**	37117.26**	214.76**	0.35**
Groups/Accessions	[48]	85.94**	276.13**	537.95**	62.83**	1.10**
Error	98	53.43	172.58	397.56	18.22	0.35

*Significant at 0.05. **Highly significant at 0.01. PH: plant height, DF: days to flowering, DM: days to maturing, FLR: flag leaf length-to-width ratio, and NT: number of tillers per hill.

**Table:** (b) Mean squares of analysis of variance for 7 traits among 50 accessions of upland rice.

Source of variation	df	NG	1000 GW	YP	NP	PL	SP	SF
Blocks	2	32.03^ns^	0.007^ns^	0.13^ns^	0.090^ns^	0.03^ns^	145.68^ns^	8.39^ns^
Accessions	49	1775.43*	155.001**	373.65**	1.92*	129.23**	5644.79**	4051.39**
Groups	[1]	3998**	160.58**	2434.58**	0.31*	41.10**	1607.47**	351.94**
Groups/Accessions	[48]	454.84**	154.88**	330.71**	1.046**	13.18**	1269.94**	318.59**
Error	98	167.46	0.115	0.199	0.346	3.78	400.53	83.21

*Significant at 0.05. **Significant at 0.01. NG: number of grains per panicle, 1000 GW: one thousand grain weight, YP: yield per pot, NP: number of panicles per hill, PL: panicle length, SP: spikelet per panicle, and SF: spikelet fertility.

**Table 5:** Genetic variance of 12 morphological characteristics.

Traits	MEAN	MSG	MSE	*σ*_*g*_^2^	*σ*_*p*_^2^	PCV (%)	GCV (%)	*h*_*B*_^2^ (%)	GA (%)
PH	136.36	1295.77	53.43	414.11	467.54	15.86	14.92	88.57	28.93
DF	84.39	3236.17	172.59	1021.2	1193.78	40.94	37.87	85.54	72.15
DM	111.55	5410.90	397.57	1671.11	2068.68	40.77	36.65	80.78	67.85
FLR	21.20	617.37	18.23	199.71	217.94	69.64	66.66	91.63	131.45
NT	3.86	2.12	0.35	0.59	0.94	25.15	19.92	62.72	32.49
NG	124.65	1775.43	167.47	535.98	703.45	21.28	18.57	76.19	33.40
1000 GW	24.06	155.00	0.12	51.62	51.74	29.89	29.86	99.77	61.44
YP	43.45	373.65	0.20	124.48	124.68	25.70	25.68	99.84	52.85
NP	3.81	1.93	0.35	0.52	0.87	24.55	19.06	60.26	30.47
PL	27.41	129.23	3.79	41.81	45.60	24.64	23.59	91.69	46.54
SP	159.14	5644.79	400.54	1748.09	2148.62	29.13	26.27	81.35	48.82
SF	79.25	4051.39	83.21	1322.73	1405.94	47.31	45.89	94.08	91.70

PH: plant height, DF: days to flowering, DM: days to maturing, FLR: flag leaf length to width ratio, NT: number of tiller per hill NG: number of grains per panicle, 1000 GW: one thousand grain weight, YP: yield per pot, NP: number of panicles per hill, PL: panicle length, SP: spikelet per Panicle, and SF: spikelet fertility, MSG: mean square of accessions, MSE: mean square of error, PCV: phenotypic coefficient of variation, GCV: genotypic coefficient of variation *h*_*B*_^2^: board sense heritability, GA: genetic advance, *σ*_*g*_^2^: genotypic variance, and *σ*_*p*_^2^: phenotypic variance.

**Table 6:** Pearson's correlation coefficient among 12 quantitative traits of upland rice.

	PH	DF	DM	FLR	NT	NG	1000 GW	YP	NP	PL	SP	SF
PH	1.00											
DF	0.77**	1.00										
DM	0.76**	0.90**	1.00									
FLR	0.48**	0.64**	0.48**	1.00								
NT	0.12	0.22	0.22	0.27	1.00							
NG	0.77**	0.93**	0.99**	0.74**	0.28	1.00						
1000 GW	0.28	0.19	0.15	0.02	−0.02	0.13	1.00					
YP	0.38**	0.31*	0.36**	0.06	0.48**	0.34*	0.34*	1.00				
NP	0.11	0.20	0.22	0.25	0.99**	0.28	−0.01	0.48**	1.00			
PL	0.46**	0.52**	0.45**	0.53**	0.14	0.51**	0.25	0.20	0.15	1.00		
SP	−0.08	−0.13	−0.12	−0.04	−0.23	−0.12	−0.28*	−0.08	−0.24	−0.10	1.00	
SF	0.39**	0.37**	0.29*	0.26	0.18	0.31*	0.23	0.26	0.18	0.26	−0.67**	1.00

*Significantly at 0.05. **Significantly at 0.01. PH: plant height, DF: days to flowering, DM: days to maturing, FLR: flag Leaf length to width ratio, NT: number of tillers per hill, NG: number of grains per panicle, 1000 GW: one thousand grain weight, YP: yield per pot, NP: number of panicles per hill, PL: panicle length, SP: spikelet per panicle, and SF: spikelet fertility.

**Table 7:** Groups of upland rice accessions according to cluster analysis.

Groups	Accessions
Group I	06040, 06041, 06048, 06068, 06070, 06067
Group II	06043, 06050, 06044, 06059, 06071, 06045, 03835, 03824, 03834, 03838
Group III	07537, 07571, 07544, 07539, 07538, 07546, 07589, 07597, 07576,07574, 07595, 03825, 03830, 07531, 07535, 07585, 07534, 03831,07509, 07540, 07543, 07590, 07594, 07588, 07575,03832, 03833
Group IV	03826, 03837
Group V	07541, 07596, 03828, 07545
Group VI	07508

**Table 8:** Mean value of 12 quantitative traits for six groups by cluster analysis on 50 upland rice accessions.

Group	PH	DF	DM	FLR	NT	NG	1000 GW	YP	NP	PL	SP	SF
I	147.97	112.87	144.00	31.12	4.50	134.36	25.99	52.42	4.40	30.06	148.30	90.70
II	142.81	88.73	121.85	19.26	3.53	141.97	26.32	48.40	3.49	27.79	164.55	86.84
III	132.04	77.65	102.40	19.59	3.71	116.66	23.71	39.10	3.68	26.97	153.28	76.68
IV	142.10	96.64	129.58	24.96	4.00	117.32	23.32	47.92	3.92	30.07	187.43	62.62
V	131.10	72.07	94.74	21.13	4.40	128.78	16.03	41.90	4.29	25.44	199.16	64.63
VI	128.06	76.89	92.28	17.31	4.75	107.03	33.03	55.13	4.67	22.20	111.70	95.86

PH: plant height, DF: days to flowering, DM: days to maturing, FLR: flag leaf length-to-width ratio, NT: number of tiller per hill NG: number of grains per panicle, 1000 GW: one thousand grain weight, YP: yield per pot, NP: number of panicles per hill, PL: panicle length, SP: spikelet per panicle, and SF: spikelet fertility.

**Table 9:** Eigenvectors and eigenvalues of the first four principal components.

Variable	Eigenvectors			
	PC 1	PC 2	PC 3	PC 4
Eigenvalue	4.37	2.14	1.53	1.14
Variation (%)	36.4	17.9	12.8	9.6
Cumulative (%)	36.4	54.5	67.1	76.7
PH	0.388	−0.227	−0.004	−0.081
DF	0.416	−0.179	0.076	0.167
DM	0.394	−0.140	0.093	0.115
FLR	0.317	−0.122	0.206	0.399
NT	0.227	0.541	0.269	−0.014
NG	0.180	−0.353	0.114	−0.473
1000 GW	0.152	0.007	−0.454	−0.288
YP	0.266	0.228	0.042	−0.591
NP	0.224	0.549	0.259	−0.017
PL	0.306	−0.135	0.008	0.197
SP	−0.141	−0.303	0.607	−0.303
SF	0.281	0.038	−0.463	−0.071

PH: plant height, DF: days to flowering, DM: days to maturing, FLR: flag leaf length to width ratio, NT: number of tillers per hill NG: number of grains per panicle, 1000 GW: one thousand grain weight, YP: yield per pot, NP: number of panicles per hill, PL: panicle length, SP: spikelet per panicle, and SF: spikelet fertility, and PC: principal components.
